# Nursing students and the internet: a reflection of digital ethics

**DOI:** 10.1590/0034-7167-2023-0459

**Published:** 2025-01-10

**Authors:** Ana Carolina Barboza Brandão, Ingrid Bemfica Ramos, Anna Carolina Caetano Griesang de Oliveira, Sabrina da Costa Machado Duarte, Joyce Martins Arimatea Branco Tavares, Priscilla Valladares Broca

**Affiliations:** I Universidade Federal do Rio de Janeiro. Rio de Janeiro, Rio de Janeiro, Brazil; II Universidade Estadual do Rio de Janeiro. Rio de Janeiro, Rio de Janeiro, Brazil

**Keywords:** Ethics, Students, Nursing, Information Technology, Internet, Education, Ética, Estudiantes de Enfermería, Tecnología de La Información, Internet, Educación

## Abstract

**Objectives:**

to identify how first-year nursing students use cyberspace and propose an orientation guide with criteria guiding the use of cyberspace.

**Methods:**

qualitative and descriptive research, carried out with 24 nursing students from a federal public institution in Rio de Janeiro. Data collection was carried out through semi-structured interviews. Data analysis occurred using IRAMUTEQ^®^. The research was approved by the institution’s Research Ethics Committee.

**Results:**

students use cyberspace to communicate, study, find “cool things”, share photos and memories. Furthermore, they are concerned about hate speech, intolerance and fake news. The good and bad sides and the types of technologies most used were also portrayed.

**Final considerations:**

the moral and ethical values of physical coexistence, together with awareness of individual responsibility, are the pillars for using cyberspace. The guide comes as an awareness tool.

## RESUMEN

**Objetivos:**

identificar cómo los estudiantes de primer año de enfermería utilizan el ciberespacio y proponer una guía de orientación con criterios que guíen el uso del ciberespacio.

**Métodos:**

investigación cualitativa y descriptiva, realizada con 24 estudiantes de enfermería de una institución pública federal de Río de Janeiro. La recolección de datos se realizó mediante entrevistas semiestructuradas. El análisis de los datos se realizó utilizando IRAMUTEQ^®^. La investigación fue aprobada por el Comité de Ética en Investigación de la institución.

**Resultados:**

los estudiantes utilizan el ciberespacio para comunicarse, estudiar, encontrar “cosas interesantes”, compartir fotografías y recuerdos. Además, les preocupan los discursos de odio, la intolerancia y las noticias falsas. También se retrataron los aspectos buenos y malos y los tipos de tecnologías más utilizadas.

**Consideraciones finales:**

los valores morales y éticos de la convivencia física, junto con la conciencia de la responsabilidad individual, son los pilares para el uso del ciberespacio. La guía viene como una herramienta de concientización.

## INTRODUCTION

In the virtual environment, there is no direct contact with others, or even the person oneis communicating with virtually does not even know and, generally, there is no concern to consider the likely consequences of actions carried out in cyberspace. In the real time and space of events, there is a direct awareness of cause-effect between action and the fruit resulting from that act^(1)^.

Cyberspace can be considered the new means of communication that arises from the global interconnection of computers, where not only the material infrastructure of digital communication is considered, but also the universe of information it hosts, as well as the human beings who use it and feed on this vast universe^(2)^.

There is still another important concept for this vast universe: cyberculture. It is considered the set of techniques, practices, attitudes, ways of thinking and values that develop along with the growth of cyberspace^(2)^. It is characterized by the social and cultural impact of the influence of new communication and information technologies on individuals’ daily lives.

From this, digital ethics becomes important to guide and regulate new “faceless” interactions, based on recognition of the other as a human being in search of interaction, information and dialogue^(3)^, having a minimum agreement that help ensure responsibility in cyberspace when it comes to information management and consumption. Digital ethics is a place of reflection whose objects are the rules and values that are implicit or explicit in this means of digital interaction^(4)^.

An important element of cyberculture that is sometimes neglected by people is e-reputation (individuals’ reputation on the virtual network)^(1)^. E-reputation is related to public information (photos or comments or opinions posted) of a person contained in the virtual world, whether information posted by the persons themselves or by third parties.

Searching for individuals’ e-reputation is also being used by human resources departments to obtain information about a job candidate and assess them regarding the vacancy profile. It is not the reputation on the network that will guarantee the job, but it can be a factor in losing it, as the candidate may have passed on information that is contradictory to that posted on the networks^(1)^.

Healthcare professionals, due to the ease of having a cell phone with a camera and quick access to the internet, have the possibility of capturing and reproducing images or situations experienced by patients at the time of care without at least asking for prior authorization from them or theirguardians^(5)^. Therefore, knowing and understanding how nursing students’ relationships with the virtual world occur and, from there, carrying out interventions can be an important strategy to avoid inappropriate situations in the future of these professionals.

Due to these types of possibilities, the Federal Nursing Council (COFEN*- Conselho Federal de Enfermagem*) launched Resolution 554^(6)^, which lists the conduct expected by nursing professionals when using social networks, with some duties, such as: the prohibition of disseminating sensational images involving professionals, patients and institutions; not being able to display the image of patients on social networks and social groups, such as WhatsApp^®^.

Undergraduate nursing students have a profile of being young, having some knowledge of IT, using the internet, having e-mail and browsing social networks, i.e., they belong to the generation that knows how to handle communication and information technologies like no one else^(7)^.

The guiding question of this work is: how do nursing students use cyberspace?

## OBJECTIVES

To identify how first-year nursing students use cyberspace and propose an orientation guide with criteria to guide the use of cyberspace by nursing students.

## METHODS

### Ethical aspects

The ethical aspects contained in Resolution 466/2012 of the Brazilian National Health Council, which deals with research involving human beings, were respected. To this end, the research project was approved by the *Escola de Enfermagem Anna Nery* and the *Instituto de Atenção à Saúde São Francisco de Assis* (EEAN/HESFA) Research Ethics Committee.

Anonymity was guaranteed, i.e., at no time were or will be the names of research participants disclosed, as agreed in the Informed Consent Form (ICF). Identification was made using alphanumeric codes, being “NS” for nursing student, followed by the sequential number of the interview.

### Study design

This is qualitative, descriptive and exploratory research. The research process followed the COnsolidated criteria for REporting Qualitative research (COREQ) step-by-step instructions.

### Study setting

The study was carried out at a nursing school at a federal institution in Rio de Janeiro. The nursing school is organized into nursing teaching departments, where professors are located by area of knowledge and lines of research. This institution has *Scricto Sensu* and *Lato Sensu* Graduate Programs, being a qualification reference for nurses across the country.

The undergraduate course curriculum is divided into eight academic periods and is organized in stages, in which meeting with patients takes place in their different moments of life, conditions and healthcare, from the first period. The choice of this institution as a research field was for convenience, since it was with these students that the problem of this research emerged.

### Data source

Nursing students from the first period participated in the study. The sample was for convenience, consisting of 24 students.

Nursing students enrolled in the institution’s first period were included. And nursing students who were away from the classroom due to illness were excluded.

Data production was interrupted from the moment the data produced were considered satisfactory, as they responded to the guiding questions and achieved the proposed objectives, therefore reaching saturation.

### Data collection and organization

Data production took place through individual interviews with a semi-structured instrument. This instrument was composed of three parts: the first contains questions with the aim of identifying participant psych sociodemographic profile; the second used free association (FA), which consists of providing stimulus words or phrases (inducing) to obtain words or phrases as responses (induced); and the third contains open-ended questions to explore the research object so that these can only be interpreted taking into account the perspective of the actors themselves.

### Work stages

Data production was carried out from October to December 2019 and followed the following steps: approach; scheduling; and the interview itself.

In the first stage, approach, the first contact was made with potential candidates for in-person participation, informing them of the objective of the research and guaranteeing the confidentiality of their identities. Once candidates were accepted, the second stage took place, in which the interview was scheduled during each person’s free time so that it did not interfere with their academic commitments. Therefore, the third stage was carried out, in which, before starting the interview, the objective of the research and anonymity were explained again, in addition to the ICF being provided.

All interviews were carried out individually on the university premises, away from places with the greatest concentration of people so that there was complete privacy. They lasted an average of 30 to 40 minutes. To carry out the interviews, the recorder contained in the researcher’s cell phone was used, with prior authorization of interviewees.

### Data analysis

The data from the first part of the data collection instrument, with the profile of nursing students, were organized and treated using simple descriptive statistics to know the percentages referring to the following characteristics: sex; age; if they have any type of information and communication technology and how many and which ones; how many times a day they use these technologies; and how many times a day they use these technologies to study or acquire knowledge.

The data from the interviews and FA were processed using the software *Interface de R pour les Analyses Multidimensionnelles de Textes et de Questionnaires*(IRAMUTEQ^®^) version 0.7 alpha 2, license 2008/2014, available on the website responsible for publishing the software (http://www.iramuteq.org/).

FA data was worked through cloud of words that grouped and organized graphically according to their frequency, as it allows quick identification of keywords in a *corpus* and, thus, obtaining a free expression of thought^(8)^. The interviews were fully transcribed into electronic text format so that their data could be processed by IRAMUTEQ^®^, which performed automatic lexical analysis on the speeches.

For the program to carry out the analysis, it was necessary to prepare the text *corpus*, where words were corrected and revised, removing language defects and capital letters present in the middle of sentences. The questions asked by the researcher were suppressed considering only participants’ responses, avoiding the use of diminutives. Furthermore, the variables that would be inserted into command lines were established and, thus, a text file (Word^®^) with eight pages was generated.

Each interview was separated by a command line composed of codes that are specific to the software. Command lines began with four asterisks (****), space and one more asterisk (*), with the identification of research participant (ind_01 to ind_24), according to the order of the interviews, in addition to space and one more asterisk (*), identifying age group (ida_1 or ida_2, where 1 = from 18 to 25 years old and 2 = from 26 to 48 years old), and space and plus asterisk (*), with identification of sex (sex_1 or sex_2, where 1= female; 2= male), as below:

*****Ind_01 *ida_1 *sex_1

With the *corpus* prepared, its processing was carried out using Descending Hierarchical Classification (DHC), which performs the type of analysis that classifies text segments (TS) according to their respective vocabularies, with the set being divided based on the frequency of reduced forms^(9)^.

From this, the software organized the data into a dendrogram, i.e., it represents the quantity and lexical composition of classes based on a grouping of terms. Afterwards, it was possible to treat the results, make inferences, interpretations, detail the interviews, understand the meaning of words, review the conceptual bases and other findings in the literature to provide a logical deduction and, thus, better analyze the findings found in the classes produced by the software.

## RESULTS

The students’ profile was important to enrich the information arising from the questions asked to students and, thus, favor a better understanding of the group. Such information was inserted into the *corpus* for analysis in the software.

Below is a simple statistical description of these data: 17 are female; and seven are male. They all have some type of information and communication technology. Thus, 22 have computers, seven have a television with a smart function, eight have a tablet and all have a cell phone. When asked how often they access such technologies to enter cyberspace, 22 responded throughout the day, one responded when necessary and responded on weekends. And when this access is to study or acquire some type of knowledge, 13 of them access it throughout the day, seven, when necessary, and four, during the night.

In relation to the DHC, it was necessary to change the default settings, leaving adverbs as supplementary words, reducing the number of classes in terminal phase 1 to eight and the TS of each class, considering lexicons with p < 0.001, result which indicates a significant association. The general *corpus* consisted of 24 texts (interviews), separated into 106 TS, with 1,010 TS used, therefore, a use of 86.18% of the material.

A total of 4,036 occurrences emerged (words, forms or words), 754 of which were distinct words and 614 words with a single occurrence (hapax). The *corpus* was divided into five classes.

**Figure 1:**
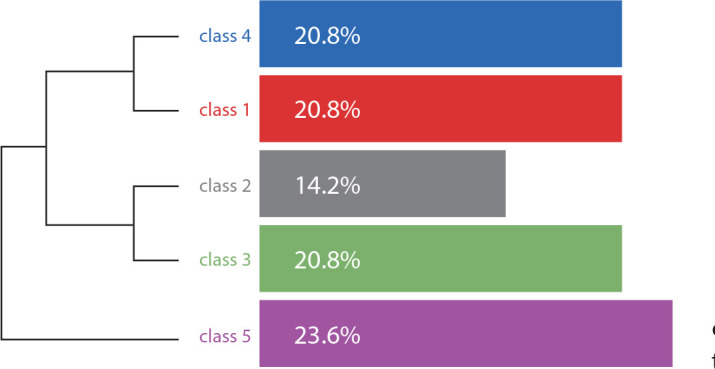
Dendrogram 1 of the Descending Hierarchical Classification

*Source: software IRAMUTEQ^®^ report, 7.2,2020.*

In relation to the dendrogram, presented horizontally, as shown in Figure 1, its reading and interpretation is done from left to right, according to the affinity between classes. It is possible to identify the partitions or iterations that were formed in TS classification in the *corpus*. These partitions generate *subcorpus* that correspond to the classes.

In Figure 1, one can identify the decreasing distribution due to TSclustering between classes, with class 5 concentrating 23.6% of TS, followed by classes 4, 3 and 1, with 20.8% each, followed by class 2, with 14.2% of TS.

From that, it is possible to observe that the *corpus* was divided (first partition) into two *subcorpus* (two thematic chunks). Thematic chunk 1 aggregates classes 4 and 1. Thematic chunk 2 aggregates classes 2 and 3. The first class to be formed or separated from the others is class 5 (second partition). The DHC ceased divisions into classes, as the five classes were composed of TS units with similar vocabulary.

As the classes do not have a conventional ascending order (1, 2, 3, 4, 5), the thematic presentation was presented considering the objectives of the study, i.e., first thematic chunk 1 and its classes were presented and, subsequently, thematic chunk 2 and its classes.

When analyzing the thematic chunks generated, it was observed that they correspond to two dimensions related to nursing students and cyberspace: “Cyberspace as a means of facilitating communication, studies and access to information” (Classes 4 and 1) and “Conduct in cyberspace” (classes 2 and 3; class 5).

### Thematic chunk 1 - Cyberspace as a means of facilitating communication, studies and access to information

In this part, the presentation of results and discussions are based on results of classes 4 and 1 and aim to analyze how nursing students use cyberspace and with what intention.

### Class 4: A place to communicate and obtain information

This class has 22 TS, accounting for 20.75% of the material classified for analysis. It is evident that this class is associated with the following variables: good (chi^2^-24.92); communicate (chi^2^-23.44); and bad (chi^2^-21.06). NS 21 stood out.

Nursing students took into account their personal experiences to present their perceptions of how they usually use the virtual world and for what purpose. In this regard, they portray two sides of this use: the good, which is to know and obtain information at any time, reach a large number of individuals and make new friends, using it for leisure and to do good for others; and the bad, which are lies, prejudice and not putting oneself in someone else’s shoes or not thinking before acting.

*The good is information, and the bad is lies and prejudice; I use it for knowledge even for entertainment.* (NS 21)

*The good thing is that it is possible to reach a greater number of people for good, such as raising money to help people, animals, social actions, NGOs and also access to information anywhere and at any time. The bad thing is that people use it individually and selfishly.* (NS 13)

### Class 1: Study environment and finding “things”

This class (1) has 22 ST, accounting for 20.75% of the material classified for analysis. It is evident that this class is associated with the following variables: thing (chi^2^-25.18); find (chi^2^-16.36); and study (chi^2^-12.06). The prominent participants in this class were interviewees NS 05, NS 16 and NS 24.

In this class, students demonstrated that they use cyberspace not only to communicate and interact with each other, but also to study, find things that, in their perception, are interesting and cool, and to know and understand a little more about the profession who chose.

*I use it a lot for research and to talk to people who are far away. The good thing is that it helps you find things you need for your daily life, and it’s bad because it ends up taking away some of the physical encounters.* (NS 5)

*To talk to my friends or study or see cool and interesting things*. [...] *Isearched for nursing to find out whether I would choose the course or not, and I found a lot of cool things, especially because I already had friends who did it, you know?*(NS 16)

*To gain knowledge, study and communicate. And I also see that the entire nursing staff is seen according to the assessments they receive from numerous sources, inducing an avalanche of baseless information and fueled by the media and prejudice*. (NS 24)

### Thematic chunk 2: Conduct in cyberspace

In this part, the presentation of results is based on results of classes 2, 3 and 5, and aims to analyze how nursing students see cyberspace, how they use information and communication technologies and their implications for ethics.

### Class 2: Nursing students in cyberspace

This class (1) has 15 TS, accounting for 14.15% of the material classified for analysis. It is evident that this class is associated with the following variables: photo (chi^2^ -25.06); issue (chi^2^ -20.24); and ethics (chi^2^ -18.73). And there was no prominent variable.

In this class, it can be observed that nursing students report in TS that they like to take and share their photos in the virtual world, but with the aim of keeping them for memory and being able to remember them in the future and be proud of what they experienced. However, it is necessary to think and act responsibly, as many act without thinking about the consequences that their actions may have in this virtual environment by not acting responsibly.

*Ethically and with respect, at the very least, because the issue is lack of supervision, because everyone does what they want without thinking. As I said before, it’s no man’s land. And the photos are for memory, to remember each stage experienced and nothing more!* (NS 2)

*More disclosure about the ethics of shared information, controlling false information, as many fall for it and follow the truth, because we must respect the laws, ethics and permission of those responsible who will have their details published, especially the photos.* (NS 4)

One can also highlight in this class a concern among nursing students with something that is increasingly present in the virtual world, which is cyberbullying. In the TS below, it is possible to observe the concern about lack of impunity, hate speech and intolerance, which are increasingly present in people’s posts.

*The bad is cyberbullying, hate speech, fake news, feelings of superiority, because there are people who want to feel superior and use the internet to do so, to humiliate another person and the internet.* (NS 1803)

*The creation of groups that have opinions against human rights, for instance, and thus the virtual world ends up increasing hatred in itself and in the real world.* (NS 07)

### Class 3: Cyberspace and fake news

This class (1) has 22 TS, accounting for 20.75% of the material. This class is associated with the following variables: fake (chi^2^-20.16); news (chi^2^-16.28); propagate; and crime (chi^2^-15.87 each). NS 15 stood out in this class.

In TS of this class, nursing students only highlight the bad side of cyberspace, which is characterized by fake news. Thus, they portray the use of the virtual world as an instrument to commit crimes through a discourse with untruths and that, perhaps, they would not do so if they were in person, live, with people.

*Excessively, because people expose themselves too much and spread false information and other people with a little less information take it as truth. So, for good, quick information and new knowledge, for bad, fake news.* (NS 15)

*There is a feeling of impunity. Many people use the internet to spread hate speech and prejudice with fake profiles, they feel encouraged to say things that they might not say in person.* (NS 11)

### Class 5: How they use communication and information technologies

This class (5) has 25 TS, accounting for 23.58% of the material classified for analysis. It is evident that this class is associated with the following variables: cell phone (chi^2^ - 84.97); long (chi^2^ - 84.85); and day (chi^2^ - 75.32). And there was no prominent variable.

In this class, nursing students report the type of technology they use to access cyberspace. In this case, they use a cell phone and computer or laptop, and connect to the virtual world throughout the day, regardless of time and location.

*I use my computer and cell phone to study and talk to people throughout the day.* (NS 1)

*I use my cell phone and computer throughout the day to read articles and watch videos on topics that I like.* (NS 22)

### Word cloud

**Figure 2:**
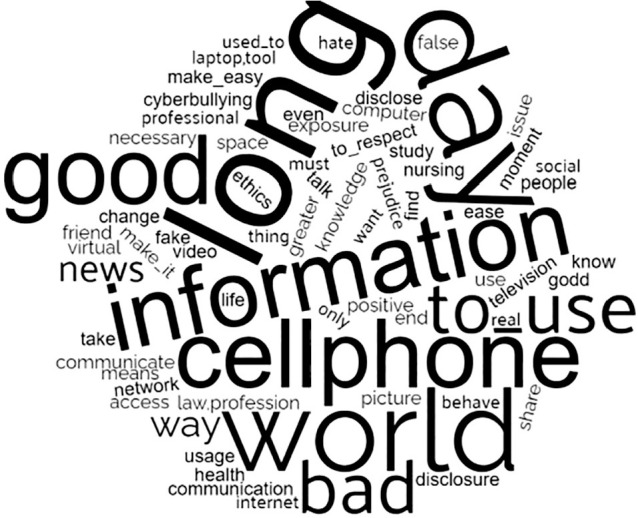
Word cloud

*Source: software IRAMUTEQ^®^ report 7.2, 2020.*

Through this analysis, it was possible to observe the importance of the word “day”, which was the most frequent in the *corpus*, 46 times, followed by the words“information”, “world” and “good”, with 40, 38 and 34 occurrences, respectively. It is possible to observe, in Figure 2, that the words are positioned randomly, however the most frequent words appear larger than the others, in order to display their prominence in the research analysis *corpus*.

**Figure 3:**
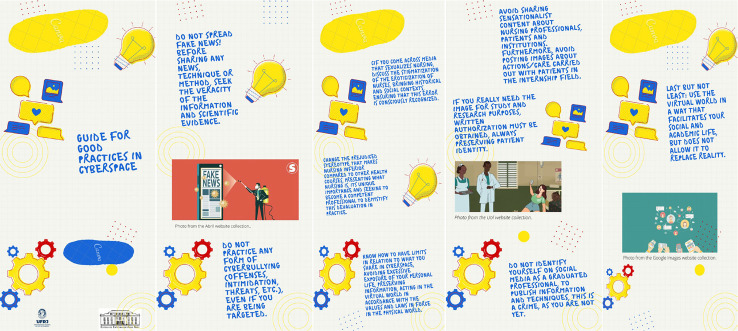
Guide for Good Practices

For the purposes of this study, after the processing steps, the meanings of these words in participants’ speeches were interpreted. Thus, the word “day” relates to the aspect of nursing students accessing cyberspace at any time of the day and in any environment in which they find themselves, for instance: “I use it whenever I have access”, “daily”, “every day”, “throughout the day”, “I donot go without a day”.

In relation to the words“information”, “world” and “good”, it was identified how pleasurable and positive students’ experience with cyberspace is. Through this environment, they can: be “whoever we want”; connect with a “diversity of people”; have “tools that make everyday life easier”; have “quick communication”; have “interaction” facilitated; exercise “freedom of expression”; between others.

### TheGuide for Good Practices

This guide was constructed based on participants’ reality, using the know-how and know-how-to-think of students and researchers, taking as a reference the conceptual bases of the researched area, with the aim of seeking and promoting transformations.

It is worth noting that this guide is not intended to limit publications or take away students’ freedom of expression, but rather to suggest that they carry out responsible, committed activities and that they become aware of the consequences that their attitudes may have on their lives and those of others other people.

The first part of the guide, which has the format of an informative folder, has its title and the symbols of EEAN and the *Universidade Federal do Rio de Janeiro* (UFRJ) to represent that it is a product created and formed within the academic environment. In the second part, students are led to reflect on fake news, in order to think and assess their veracity before sharing any information and about not practicing and/or encouraging cyberbullying. In the third part, they are made aware not to take photos or post their actions and care as a nursing student and, if necessary, for academic purposes, there is the need to have a patient authorization form.

In the fourth part, students are led to think about how nursing is seen in the virtual world and encouraged to always disseminate information that demonstrates the importance of the profession. And they are also encouraged to avoid making major exposures of their lives on social media and always act in accordance with the moral values and laws in force in the world outside the virtual world. And in the last part, the following message is left: “use the virtual world in a way that facilitates your social and academic life, but does not allow it to replace reality”.

## DISCUSSION

In relation to nursing students’ sex, such findings are in line with the entire historical trajectory of the profession of being mostly female^(10)^. But it is worth highlighting that, even though it is a mostly female profession, they consider that in recent years there has been a tendency for male participation in the category to increase^(10)^.

People use the virtual world as a tool to talk, relate and interact, whether with individuals they already know or with those they know through social media, but do not have face-to-face interaction. The opportunities offered by the digital world are impressive, but it is important not to absolutize them theoretically or practically, and to do so requires thought and time, and thinking takes time^(11)^.

Cyberspace allows social interaction, the sharing of values, knowledge, objectives, opportunities, connecting people, providing new friendships and allowing rapid mobilization between individuals anywhere in the world. Currently, the use of this medium by healthcare professionals provides a new ethical context in training and care practice, as most people have a camera, which can easily record images and videos of situations experienced by patients, co-workers or in practice academic within healthcare institutions^(12)^.

The use of cyberspace is gradually increasing among nursing students and professionals, because it is a space for social interaction, where health information can be obtained and shared, as it facilitates communication and allows exchange of opinions and experiences from home or abroad work. Furthermore, they facilitate updating, as it is a fundamental tool for biomedical journals to disseminate their scientific articles to researchers and readers^(13)^.

Nursing students, who are mostly young, use the virtual world as a tool to produce and post their ideas, thoughts and promote many ways of interaction and socialization^(14)^. Within this use, photographs of patients may be displayed during an academic activity in health services, as was identified in research carried out with university health students in Manaus, without requesting permission to do so^(12)^. And in research carried out in Mexico, nursing professionals are not respecting patients’ confidentiality when publishing images and information^(13)^.

Considering such information, it is necessary to make use of ethics of care, which are aspects of ethics and morals applied in health, such as empathy, autonomy (of professionals and patients), commitment to human rights and duties, citizenship and multiculturalism^(15)^.

The relationships established in the virtual world cross the borders of nations and, even so, the rights and duties in this environment cannot be isolated from the rights and duties of the physical world^(16)^. The rules of good coexistence and safety that we use in real life also need to be used in the virtual world, i.e., common sense and thinking several times before sharing any information in the virtual world are necessary.

Therefore, nursing professionals need to maintain and define an ethical attitude when publishing or sharing information related to patients’ health^(13)^. Therefore, ethics proves to be an unfolding of the social interactions of the closest people, from which affective bonds are established, and moral behavior would have as its foundation the reciprocity present, mainly, in care relationships^(17)^.

Privacy in cyberspace has no longer been a priority among people, as healthcare professionals, including students, even with authorization, can unnecessarily expose information and images of patients^(12)^. Consequently, they do not consider the criteria established by the Nursing Code of Ethics and COFEN resolution.

Furthermore, the virtual world is neither closed nor private, and a photo can conquer the world in seconds when viewed by millions of people, as it is not protected behind a computer screen. Therefore, it is possible to consider and imagine a future in which the difference between the real and the digital, as a possible perspective on life, is perceived as confusing^(11)^. Therefore, data disclosed in the virtual world involves a risk, because anyone can download or share information and, consequently, remain online for an indefinite period of time; thus, information that is shared or published may cause an ethical or legal problem^(18)^.

During their educational training, nursing students acquire a professional identity that carries values and behaviors and that guarantees patients’ trust; therefore, it is necessary to be careful with information that is published in the virtual world so as not to create a negative image of work^(18)^. Such values and behaviors can revolve around the care received, especially during childhood, involving feelings and affections that can serve as a basis for what is interpreted as “right” or “wrong” in human relationships, which can be considered as “care about”^(17)^.

It is necessary not only to think about the workplace, but also about the interpersonal relationships between colleagues, as a photo, a video or a comment shared in the virtual world can cause a conflict between them and make daily coexistence difficult and, therefore, affect the progress of assistance and even lead to their dismissal. Therefore, students need to become aware from the moment they graduate of the importance of respecting the rules of good coexistence also in cyberspace so that there are no disagreements among co-workers and this affects the care offered.

When sharing ideas, values and thoughts in the virtual world opens up space for hate speech and intolerance, with freedom of expression and free will as justification, it cannot be considered an argument. Such offenses define bullying, which has already affected 27% of children and young people between 9 and 17 years old in the country in the last 12 months^(14)^. Offensive content, with the sole intention of targeting specific groups, such as social minorities, is always aimed at the inferiorization of a person, race, gender, ethnicity, nationality, religion, sexual orientation, or other aspect, in the virtual world, characterizing cyberbullying^(18)^.

Cyberbullying is related to using communication and information technologies to threaten, humiliate or carry out any action with malicious intent towards someone, which may affect coexistence, victims’ mental well-being and their rights^(14)^. Generally, it aims to affect individuals’ psychological and emotional aspects, but it can lead to physical violence itself.

No matter how much a person clarifies their behavior and the reason that led them to spread this speech of hate and intolerance, it is something difficult to forget. Even more so with the speed at which information spreads in the virtual world, the topic can always be remembered and encourage new reactions.

Therefore, it is necessary to have good management of what is shared or posted in cyberspace, to avoid embarrassment or even behavior that directly affects the profession and/or work environment and/or the position one holds at the time and/or personal life. It is necessary to verify the veracity of the information shared, especially involving health issues, as profiles have been identified that publish false information or that come from unreliable sources, a situation that can cause health risks to the followers of these pages^(13)^.

Therefore, it is necessary to train nursing professionals who are committed to disclosing digital information, which involves some regulatory, legal and moral considerations against academic dishonesty, such as omission of citations, invention of content, self-plagiarism, duplication of information, unauthorized copies source code and false authorship. Such content must be emphasized as a fundamental axis for the profession^(16,18)^.

Furthermore, people and society in general need to be legally protected, but not in an excessive and paternalistic way, nor subject to total control without any type of legal agreement on the needs and limits of such measures^(19)^. People can make use of moral obligation, which involves recognizing other individuals as similar. With regard to the need to give and receive care, the real feelings for the other apply indirectly^(17)^.

In this way, the guide can help nursing students to better understand these examples, even during their undergraduate studies, to prevent such actions from being replicated when they are in the job market (and also in their personal lives). Therefore, nursing professionals must be equipped with solid ethical and technological experience, which must begin at the university stage, with the aim of respecting ethical principles and, thus, improving the ability to prevent and resolve ethical conflicts in the performance of their role^(18)^.

Hence, this professional and academic practice can be guided by ethics of care, which can be represented by the synthesis of moral values applied to care practice, covering moral virtues, prudence, justice, respect for autonomy, freedom and democracy^(15)^.

### Study limitations

The limitation of this study is that interviews were carried out only at one university and with only the first period class, which limits an analysis of the Brazilian reality. The inclusion of other universities may expand the scope of analysis of nursing students’ behavior in cyberspace.

### Contributions to nursing, health or public policy

The present study encourages students to be careful with what is posted and shared in cyberspace, as information may be untrue, without any control and without being able to identify the original source. Therefore, they need to stop to reflect and analyze calmly and within ethical precepts what they are reading, posting and sharing on their virtual networks.

## FINAL CONSIDERATIONS

Nursing students use cyberspace to facilitate communication, interact, study, obtain information, store photos and files, but also portray that bad actions can be identified, such as fake news and cyberbullying.

In this sense, it is important to include and/or reinforce in the nursing course curriculum how to best use cyberspace, highlighting ethical and legal principles as well as the impact on their professional and personal life. Thus, the guidance guide could be an important tool to guide these actions with educational and healthcare institutions.
