# Supplementary material for: Nursing students and the internet: a reflection of digital ethics
Source: Rev Bras Enferm. 2025 Jan 10;77(Suppl 4):e20230459. doi: 10.1590/0034-7167-2023-0459 (PMC11726820; doi:10.1590/0034-7167-2023-0459)
Supplement: Translation

# Translation: Os estudantes de enfermagem e a internet: uma reflexão da ética digital

**DOI:** 10.1590/0034-7167-2023-0459pt

**Authors:** Ana Carolina Barboza Brandão, Ingrid Bemfica Ramos, Anna Carolina Caetano Griesang de Oliveira, Sabrina da Costa Machado Duarte, Joyce Martins Arimatea Branco Tavares, Priscilla Valladares Broca

**Affiliations:** I Universidade Federal do Rio de Janeiro. Rio de Janeiro, Rio de Janeiro, Brasil; II Universidade Estadual do Rio de Janeiro. Rio de Janeiro, Rio de Janeiro, Brasil

**Keywords:** Ética, Estudantes de Enfermagem, Tecnologia da Informação, Internet, Educação

## Abstract

**Objetivos:**

identificar como acadêmicos de enfermagem do primeiro período usam o ciberespaço e propor um guia de orientação com critérios norteadores do uso do ciberespaço.

**Métodos:**

pesquisa qualitativa e descritiva, realizada com 24 acadêmicos de enfermagem de instituição pública federal do Rio de Janeiro. A coleta de dados foi realizada através de entrevista semiestruturada. Análise dos dados ocorreu pelo IRAMUTEQ^®^. A pesquisa foi aprovada pelo Comitê de Ética em Pesquisa da instituição.

**Resultados:**

os estudantes usam o ciberespaço para se comunicar, estudar, achar “coisas legais”, compartilhar fotos e memórias. Além disso, preocupam-se com discursos de ódio, intolerância e *fake news.* Também foram retratados o lado bom e o ruim e os tipos de tecnologias mais usadas.

**Considerações finais:**

os valores morais e éticos da convivência física, juntamente com a consciência de responsabilidade individual, são os pilares para o uso do ciberespaço. O guia vem como um instrumento de conscientização.

## INTRODUÇÃO

No ambiente virtual, não há um contato direto com o outro, ou até mesmo nem se conhece a pessoa com quem se comunica virtualmente e, geralmente, não há uma preocupação em considerar as prováveis sequelas das ações realizadas no ciberespaço. No tempo e espaço real dos acontecimentos, há uma consciência direta de causa-efeito entre ação e o fruto decorrente desse ato^(1)^.

O ciberespaço pode ser considerado o novo meio de comunicação que surge da interconexão mundial dos computadores, onde não é considerada apenas a infraestrutura material da comunicação digital, mas também o universo de informações que ela hospeda, assim como os seres humanos que a utilizam e se alimentam desse vasto universo^(2)^.

Ainda há outro importante conceito para este vasto universo: a cibercultura. Ela é considerada o conjunto de técnicas, práticas, atitudes, modos de pensamento e valores que se desenvolvem juntamente com o crescimento do ciberespaço^(2)^. É caracterizada pelo impacto social e cultural da influencia das novas tecnologias da comunicação e informação no cotidiano dos indivíduos.

A partir disso, a ética digital se faz importante para guiar e regular as novas interações “sem rosto”, tendo como base o reconhecimento do outro como um ser humano em busca de interação, informação e diálogo^(3)^, tendo um acordo mínimo que ajude a garantir a responsabilidade no ciberespaço no que diz ao manejo e consumo de informação.A ética digital é um local de reflexão cujos objetos são as regras e valores que estão implícitos ou explícitos nesse meio de interação digital^(4)^.

Um importante elemento da cibercultura que às vezes é negligenciado pelas pessoas é a *e-reputation* (a reputação do individuo na rede virtual)^(1)^. A *e-reputation* está relacionada às informações públicas (fotos ou comentários ou opiniões postadas) de uma pessoa contida no mundo virtual, sejam informações postadas pela própria pessoa ou por terceiros.

A pesquisa pela *e-reputation* do indivíduo também está sendo utilizada pelos departamentos de recursos humanos para obter informações do candidato ao emprego e avaliá-lo quanto ao perfil da vaga. Não é a reputação na rede que irá garantir o emprego, mas pode ser um indutor da perda do mesmo, pois o candidato pode ter repassado informações que são contraditórias daquelas postadas nas redes^(1)^.

Os profissionais de saúde, pela facilidade de terem um aparelho celular com câmera fotográfica e acesso rápido à internet, têm a possibilidade de capturar e reproduzir imagens ou situações vivenciadas pelo paciente no momento do atendimento sem ao menos pedir uma autorização previa a ele ou ao seu responsável^(5)^. Desse modo, conhecer e entender como se dão as relações dos estudantes de enfermagem com o mundo virtual e, a partir daí, realizar intervenções pode ser uma importante estratégia para evitar situações inapropriadas no futuro desses profissionais.

Devido a esses tipos de possibilidades, o Conselho Federal de Enfermagem (COFEN) lançou a Resolução n^o^. 554^(6)^, que lista as condutas esperadas pelos profissionais de enfermagem ao utilizarem as redes sociais, tendo alguns deveres, como: a proibição de divulgar imagens sensacionalistas envolvendo profissionais, pacientes e instituições; não poder expor a imagem de pacientes em redes sociais e grupos sociais, tais como o *WhatsApp^®^*.

Os estudantes de graduação de enfermagem possuem um perfil de ser jovens, ter algum conhecimento em informática, utilizar a internet, possuir *e-mail* e navegar nas redes sociais, ou seja, pertencem à geração que sabe manipular como ninguém as tecnologias da comunicação e informação^(7)^.

A questão norteadora do trabalho é: como os acadêmicos de enfermagem usam o ciberespaço?

## OBJETIVOS

Identificar como os acadêmicos de enfermagem do primeiro período usam o ciberespaço e propor um guia de orientação com critérios norteadores do uso do ciberespaço pelos acadêmicos de enfermagem.

## MÉTODOS

### Aspectos éticos

Foram respeitados os aspectos éticos contidos na Resolução n^o^. 466/2012 do Conselho Nacional de Saúde, que trata de pesquisas envolvendo seres humanos. Para isso, o projeto de pesquisa foi aprovado pelo Comitê de Ética e Pesquisa da Escola de Enfermagem Anna Nery e do Instituto de Atenção à Saúde São Francisco de Assis (EEAN/HESFA).

O anonimato foi garantido, ou seja, em nenhum momento foram ou serão divulgados os nomes dos participantes da pesquisa, conforme acordado no Termo de Consentimento Livre e Esclarecido (TCLE). A identificação foi feita por códigos alfanuméricos, sendo “AE” para acadêmico de enfermagem, seguida do número sequencial da entrevista.

### Tipo de estudo

Trata-se de pesquisa qualitativa, descritiva e exploratória. O processo de pesquisa seguiu o passo a passo do *COnsolidated criteria for REporting Qualitative research* (COREQ).

### Cenário do estudo

O estudo foi realizado em escola de enfermagem de instituição federal do Rio de Janeiro. A escola de enfermagem se organiza em departamentos de ensino de enfermagem, onde se localizam os professores por área de conhecimento e linhas de pesquisa. Essa instituição possui Programas de Pós-Graduação *Scricto Sensu*e *Lato Sensu*, sendo referência de qualificação para enfermeiros de todo o país.

O currículo do curso de graduação é dividido em oito períodos letivos e se organiza em etapas, no qual o encontro com os pacientes acontece em seus diferentes momentos de vida, condições e atenção à saúde, desde o primeiro período. A escolha dessa instituição como campo de pesquisa foi por conveniência, uma vez que foi com esses estudantes que emergiu a problemática desta pesquisa.

### Fonte de dados

Participaram do estudo acadêmicos de enfermagem do primeiro período. A amostra foi por conveniência, sendo composta por 24 alunos.

Foram incluídos acadêmicos de enfermagem inseridos no primeiro período da instituição. E foram excluídos acadêmicos de enfermagem que estiveram afastados da sala de aula por motivos de doença.

A produção de dados foi interrompida a partir do momento que se consideraram satisfatórios os dados produzidos, já que esses respondiam às questões norteadoras e alcançaram os objetivos propostos, alcançando, portanto, a saturação.

### Coleta e organização dos dados

A produção dos dados se deu através de entrevista individual com um instrumento semi-estruturado. Esse instrumento foi composto de três partes: na primeira, constam perguntas com o intuito de captar o perfil psicosociodemográfico dos participantes; na segunda, foi utilizada a livre associação de ideias (LAI), que consiste no fornecimento de palavras ou frases de estímulo (indutoras) para se obter palavras ou frases como respostas (induzidas); e na terceira, constam questões abertas para exploração do objeto da pesquisa, de modo que essas só podem ser interpretadas levando em conta a perspectiva dos próprios atores.

### Etapas do trabalho

A produção de dados foi realizada no período de outubro a dezembro de 2019 e seguiu as seguintes etapas: abordagem; agendamento; e a entrevista propriamente dita.

Na primeira etapa, abordagem, foi feito o primeiro contato com potenciais candidatos para participação presencial, informando-os do objetivo da pesquisa e garantindo o sigilo de suas identidades. Com a aceitação dos candidatos, seguiu-se para a segunda etapa, na qualfoi marcada a entrevista durante o horário livre de cada um, de modo que não interferisse em seus compromissos acadêmicos. Sendo assim, foi realizada a terceira etapa, na qual, antes de iniciar a entrevista, foram explicados novamente o objetivo da pesquisa e o anonimato, além de ter sigo entregue o TCLE.

Todas as entrevistas foram realizadas individualmente nas dependências da universidade, afastadas dos locais de maior concentração de pessoas, para que houvesse total privacidade. As mesmas tiveram duração em média de 30 a 40 minutos. Para a realização das entrevistas, foi utilizado o gravador contido no telefone celular da pesquisadora, com a prévia autorização dos entrevistados.

### Análise dos dados

Os dados provenientes da primeira parte do instrumento de coleta de dados, com o perfil dos acadêmicos de enfermagem, foram organizados e tratados a partir de estatística simples descritiva para conhecer os percentuais referentes às seguintes características: sexo; idade; se possui algum tipo de tecnologia de informação e comunicação e quantas e quais; quantas vezes ao dia utilizam essas tecnologias; e quantas vezes ao dia utilizam essas tecnologias para estudar ou adquirir conhecimento.

Os dados provenientes das entrevistas e da LAI foram trabalhados através do *software Interface de R pour les Analyses Multidimensionnelles de Textes et de Questionnaires* (IRAMUTEQ^®^), versão 0,7 alpha 2, licença 2008/2014, disponível no site responsável pela divulgação do *software* (http://www.iramuteq.org/).

Os dados da LAI foram trabalhados através da nuvem de palavras que agrupou e organizou graficamente em função da sua frequência, na medida em que possibilita rápida identificação das palavras-chave de um *corpus* e, assim, obter uma expressão livre do pensamento^(8)^. As entrevistas foram integralmente transcritas em formato de texto eletrônico, para que seus dados pudessem ser processados pelo IRAMUTEQ^®^, que fez a análise lexical automática nos discursos.

Para o programa realizar a análise, foi necessário realizar uma preparação do *corpus* textual, onde é feita uma correção e revisão das palavras, retirando os vícios de linguagem e as letras maiúsculas presentes no meio das frases. As perguntas realizadas pela pesquisadora foram suprimidas considerando-se apenas as respostas dos participantes, evitando-se o uso de diminutivos. Além disso, foram estabelecidas as variáveis que seriam inseridas nas linhas de comando e, assim, gerou-se um arquivo de texto (Word^®^) com oito páginas.

Cada entrevista foi separada por uma linha de comando composta por códigos que são específicos do *software*. As linhas de comando iniciaram-se por quatro asteriscos (****), espaço e mais um asterisco (*), com a identificação do participante da pesquisa (ind_01 até ind_24), conforme ordem das entrevistas, além de espaço e mais um asterisco (*), com a identificação da faixa etária (ida_1 ou ida_2, em que1= de 18 a 25 anos e 2= de 26 a 48 anos), e espaço e mais asterisco (*), com a identificação do sexo (sex_1 ou sex_2, onde 1= feminino; 2= masculino), conforme abaixo:

*****Ind_01 *ida_1 *sex_1

Com o *corpus* preparado, foi realizado o seu processamento através da Classificação Hierárquica Descendente (CHD), que faz o tipo de análise que classifica os segmentos de textos (ST) em função dos seus vocabulários respectivos, sendo seu conjunto repartido com base na frequência das formas reduzidas^(9)^.

A partir disso, o *software* organizou os dados em um dendrograma, isto é, ele representa a quantidade e a composição léxica de classes a partir de um agrupamento de termos. Após, foi possível tratar os resultados, fazer inferências, interpretações, detalhar as entrevistas, compreender o significado das palavras, retomar as bases conceituais e outros achados na literatura para proporcionar uma dedução lógica e, assim, melhor analisar os achados encontrados nas classes produzidas pelo *software*.

## RESULTADOS

O perfil dos estudantes foi importante para enriquecer as informações provenientes das perguntas realizadas aos alunos e, assim, favorecer uma melhor compreensão do grupo. Tais informações foram inseridas no *corpus* para análise no *software*.

A seguir, segue a descrição estatística simples desses dados: 17 são do sexo feminino; e sete são do sexo masculino. Todos eles possuem algum tipo de tecnologia da informação e comunicação. Assim, 22 possuem computadores, sete possuem televisão com função *smart*, oito possuem *tablet* e todos contêm aparelho celular. Ao serem questionados quantas vezes acessam tais tecnologias para entrar no ciberespaço, 22 responderam ao longo do dia, um respondeu quando necessário e respondeu aos fins de semana. E quando esse acesso é para estudar ou adquirir algum tipo de conhecimento, 13 deles acessam ao longo do dia, sete, quando necessário, e quatro, durante a noite.

Em relação à CHD, foi preciso alterar as configurações padrão, deixando advérbios como palavra suplementar, reduzir o número de classes na fase terminal 1 para oito e os ST de cada classe, considerando os léxicos com p < 0,001, resultado que aponta associação significativa. O *corpus* geral foi constituído por 24 textos (entrevistas), separados em 106 ST, com 1.010 ST aproveitados, portanto, um aproveitamento de 86,18 % do material.

Emergiram 4.036 ocorrências (palavras, formas ou vocábulos), sendo 754 palavras distintas e 614 palavras com uma única ocorrência (hápax). O *corpus* foi dividido em cinco classes.

Em relação ao dendrograma, apresentado na forma horizontal, conforme a Figura 1, sua leitura e interpretação é feita da esquerda para a direita, conforme a afinidade entre as classes. É possível identificar as partições ou iterações que foram formadas na classificação dos ST do *corpus*. Essas partições geram *subcorpus* que correspondem às classes.

**Figura 1:** Dendrograma 1 da Classificação Hierárquica Descendente

*Fonte:relatório do software IRAMUTEQ®, 7.2,2020.*

Na Figura 1, pode-se identificar a distribuição decrescente por aglutinação de ST entre as classes, sendo que a classe 5 concentra 23,6% dos ST, seguida das classes 4, 3 e 1, com 20,8% cada, seguida da classe 2, com 14,2% dos ST.

A partir disso, é possível observar que o *corpus* foi dividido (primeira partição) em dois *subcorpus* (dois blocos temáticos). O bloco temático 1 agrega as classes 4 e 1. O bloco temático 2 agrega as classes 2 e 3. A primeira classe a ser formada ou separada das demais é a classe 5 (segunda partição). A CHD cessou as divisões em classes, pois as cinco classes foram compostas de unidades de ST com vocabulário semelhante.

Como as classes não possuem uma ordem crescente convencional (1, 2, 3, 4, 5), a apresentação temática foi apresentada considerando os objetivos do estudo, ou seja, primeiramente foram apresentados o bloco temático 1 e suas classes e, na sequência, o bloco temático 2 e suas classes.

Ao analisar os blocos temáticos gerados, observou-se que esses correspondem a duas dimensões relacionadas aos acadêmicos de enfermagem e ciberespaço: “O ciberespaço como meio de facilitar a comunicação, os estudos e o acesso à informação” (Classes 4 e 1) e “Condutas no ciberespaço” (classes 2 e 3; classe 5).

### Bloco temático 1 - O ciberespaço como meio de facilitar a comunicação, os estudos e o acesso à informação

Nesta parte, a apresentação dos resultados e as discussões se baseiam nos resultados das classes 4 e 1, e têm como finalidade analisar como os acadêmicos de enfermagem fazem uso do ciberespaço e com que intenção.

### Classe 4: Um lugar para se comunicar e obter informações

Essa classe conta com 22 ST, representando 20,75% do material classificado para análise. Evidencia-se que essa classe possui associação com as seguintes variáveis: bom (chi^2^-24.92); comunicar (chi^2^-23.44); e ruim (chi^2^-21.06). O participante de destaque foi o AE 21.

Os acadêmicos de enfermagem levaram em consideração suas experiências pessoais para apresentar suas percepções de como costumam utilizar o mundo virtual e com qual finalidade. Desse modo, eles retratam dois lados desse uso: o bom, que é para conhecer e obter informações a qualquer momento, alcançar um grande número de indivíduos e fazer novos amigos, usando para o lazer e para fazer o bem ao próximo; e o ruim, que são as mentiras, o preconceito e o não se colocar no lugar do outro ou não pensar antes de agir.

*De bom, tem a informação, e de ruim, tem a mentira e o preconceito; eu uso pra conhecimento mesmo pra entretenimento.* (AE 21)

*De bom, é possível alcançar um número maior de pessoas para o bem, como arrecadação de dinheiro para ajudar pessoas, animais, ações sociais, ONG se também o acesso à informação em qualquer lugar e a qualquer momento. De ruim, as pessoas a utilizam de forma individual e egoísta* (AE 13)

### Classe 1: Ambiente de estudos e achar “coisas”

Essa classe (1) conta com 22 ST, representando 20,75% do material classificado para análise. Evidencia-se que essa classe possui associação com as seguintes variáveis: coisa (chi^2^-25.18); achar (chi^2^-16.36); e estudar (chi^2^-12.06). Os participantes de destaque nessa classe foram os entrevistados AE 05, AE 16 e AE 24.

Nessa classe, os estudantes evidenciaram que usam o ciberespaço para além de se comunicar e interagir uns com os outros, mas também para estudar, achar coisas que, em suas percepções, são interessantes e legais, e conhecer e entender um pouco mais sobre a profissão que escolheram.

*Uso bastante para pesquisas e para conversar com pessoas que estão longe. De bom, é a ajuda em achar coisas que você precisa para o seu dia a dia, e é ruim porque acaba tirando um pouco dos encontros físicos.* (AE 5)

*Para falar com meus amigos ou estudar ou ver coisas legais e interessantes.* [...] *fui pesquisar sobre a enfermagem pra saber se eu iria escolher o curso ou não, eu achei muita coisa legal, até porque eu já tinha amigas que faziam, sabe?* (AE 16)

*Para obter conhecimento, estudar e me comunicar. E também vejo que todo o corpo da enfermagem é visto de acordo com as avaliações que recebem por inúmeras fontes, induzindo uma avalanche de informações sem base e fomentada pela mídia e preconceito*. (AE 24)

### Bloco temático 2: Condutas no ciberespaço

Nessa parte, a apresentação dos resultados se baseia nos resultados das classes 2, 3 e 5, e tem como finalidade analisar como os acadêmicos de enfermagem veem o ciberespaço, como fazem uso das tecnologias da informação e comunicação e suas implicações para a ética.

### Classe 2: Os acadêmicos de enfermagem no ciberespaço

Essa classe (1) conta com 15 ST, representando 14,15% do material classificado para análise. Evidencia-se que essa classe possui associação com as seguintes variáveis: foto (chi^2^ -25.06); questão (chi^2^ -20.24); e ética (chi^2^ -18.73). E não houve variável de destaque.

Nessa classe, pode-se observar que os acadêmicos de enfermagem relatam nos ST que gostam de tirar e compartilhar suas fotos no mundo virtual, mas com o intuito de deixar guardados para a memória e poder relembrar no futuro e se orgulhar daquilo que vivenciaram. Porém, é preciso pensar e agir com responsabilidade, pois muitos agem sem pensar nas consequências que seus atos podem ter nesse ambiente virtual ao não agir com responsabilidade.

*Com ética e com respeito, no mínimo, porque a questão é a falta de fiscalização, porque todo mundo faz o que quiser sem pensar. Como eu disse antes, é terra de ninguém. E as fotos são para ter memória, pra guardar de lembrança cada etapa vivida e nada mais!* (AE 2)

*Mais divulgação sobre a ética de informações compartilhadas, controlando sobre as informações falsas, pois muitos caem e seguem à vero, porque devemos respeitar as leis, a ética e permissão dos responsáveis que terão suas particularidades publicadas, principalmente as fotos.* (AE 4)

Também pode-se destacar nessa classe uma preocupação dos acadêmicos de enfermagem com algo que está a cada dia mais presente no mundo virtual, que é o *cyberbullying.* Nas ST abaixo, é possível observar a preocupação com a falta de impunidade, com o discurso de ódio e a intolerância, que estão cada vez presentes nas postagens das pessoas.

*De ruim, o cyberbullying, discursos de ódio, fake news, sentimento de superioridade, porque há pessoas que querem se sentir superior e usam da internet para isso, para humilhar outra pessoa e a internet.* (AE 1803)

*A criação de grupos que tenham opiniões contra direitos humanos, por exemplo, e assim o mundo virtual acaba aumentando o ódio nele mesmo e no mundo real.* (AE 07)

### Classe 3: O ciberespaço e as *fake news*

Essa classe (1) conta com 22 ST, representando 20,75% do material. Essa classe possui associação com as seguintes variáveis: *fake* (chi^2^-20.16); *news* (chi^2^-16.28); propagar; e crime (chi^2^-15.87 cada uma). O participante de destaque nessa classe foi o entrevistado AE 15.

Nas ST dessa classe, os acadêmicos de enfermagem evidenciam somente o lado ruim do ciberespaço, que é caracterizado pelas *fake news*. Desse modo, retratam o uso do mundo virtual como instrumento para cometer crimes através de um discurso com inverdades e que, talvez, não o fariam se estivessem presencialmente, ao vivo, com pessoas.

*De modo excessivo, porque as pessoas se expõem demais e propagam informações falsas e outras pessoas com um pouco menos de informação levam como verdade. Então, de bom, informações rápidas e novos conhecimentos, de ruim, fake news.* (AE 15)

*Há uma sensação de impunidade. Muitas pessoas se utilizam da internet pra propagar discursos de ódio e preconceito com perfis fakes, se sentem encorajados a falar coisas que talvez ao vivo não falassem.* (AE 11)

### Classe 5: Como fazem uso das tecnologias de comunicação e informação

Essa classe (5) conta com 25 ST, representando 23, 58% do material classificado para análise. Evidencia-se que essa classe possui associação com as seguintes variáveis: celular (chi^2^ - 84.97); longo (chi^2^ - 84.85); e dia (chi^2^ - 75.32). E não houve variável de destaque.

Nessa classe, os acadêmicos de enfermagem relatam qual o tipo de tecnologia que utilizam para acessar o ciberespaço. Nesse caso, eles utilizam aparelho celular e computador ou *notebook*, e se conectam ao mundo virtual ao longo do dia, independentemente do horário e local.

*Utilizo pelo computador e celular para estudar e conversar com as pessoas ao longo do dia.* (AE 1)

*Uso o celular e o computador, ao longo do dia, para leitura de artigos e assistir vídeos sobre assuntos que eu gosto.* (AE 22)

### Nuvem de palavras

Através dessa análise, foi possível observar a importância da palavra “dia”, que foi a que teve maior frequência no *corpus,* 46 vezes, seguida das palavras “informação”, “mundo” e “bom”, com 40, 38 e 34 ocorrências, respectivamente. É possível observar, na Figura 2, que as palavras são posicionadas aleatoriamente, contudo as palavras mais frequentes aparecem maiores que as outras, de modo a exibir o seu destaque no *corpus* de análise da pesquisa.

**Figura 2:** Nuvem de palavra

*Fonte: relatório do software IRAMUTEQ® 7.2, 2020.*

**Figura 3:** Guia de Boas Práticas

Para fins deste estudo, após as etapas de processamento, foram interpretados os sentidos dessas palavras nos discursos dos participantes. Dessa forma, a palavra “dia” relaciona-se ao aspecto de os acadêmicos de enfermagem acessarem o ciberespaço a qualquer momento do dia e em qualquer ambiente em que se encontram, por exemplo: “utilizo sempre que tenho acesso”, “diariamente”, “todos os dias”, “ao longo do dia”, “não fico sem um dia”.

Em relação às palavras “informação”, “mundo” e “bom”, identificou-se o quanto é prazeroso e positivo a experiência dos acadêmicos com o ciberespaço. Através desse ambiente, eles podem: ser “quem a gente quiser”; se conectar com uma “diversidade de pessoas”; possuir “ferramentas que facilitam o dia a dia”; ter uma “rápida comunicação”; ter “interação” facilitada; exercer a “liberdade de expressão”; entre outros.

### O Guia de Boas Práticas

O presente guia foi construído a partir da realidade dos participantes utilizando o saber-fazer e o saber-pensar dos estudantes e das pesquisadoras, tendo como referência as bases conceituais da área pesquisada, com o intuito de buscar e promover transformações.

Vale ressaltar que o presente guia não tem a intenção de limitar as publicações ou tirar a liberdade de expressão dos alunos, mas sim sugerir que se tenham atividades responsáveis, comprometidas e que tomem consciência das consequências que suas atitudes podem ocasionar em suas vidas e nas de outras pessoas.

Na primeira parte do guia, que tem o formato de um *folder* informativo, tem-se o seu título e os símbolos da EEAN e da Universidade Federal do Rio de Janeiro (UFRJ) para representar que é um produto criado e formado dentro do meio acadêmico. Na segunda parte, o aluno é levado a refletir sobre as *fakes news,* de modo a pensar e avaliar a veracidade antes de compartilhar qualquer informação e sobre não praticar e/ou incentivar o *cyberbullying*. Na terceira parte, ele é levado a tomar consciência para não tirar fotos e nem postar suas ações e cuidados como estudante de enfermagem e, se precisar, para fins acadêmicos, há a necessidade de ter um termo de autorização do paciente.

Na quarta parte, o aluno é levado a pensar sobre como a enfermagem é vista no mundo virtual e incentivado a sempre divulgar informações que demonstrem a importância da profissão. E também é incentivado a evitar fazer grandes exposições de sua vida nas redes sociais e sempre agir conforme os valores morais e as leis vigentes no mundo fora do virtual. E na ultima parte, é deixado o seguinte recado: “utilize o mundo virtual de forma a facilitar sua vida social e acadêmica, e não permita que ela substitua a vida real”.

## DISCUSSÃO

Em relação ao sexo dos acadêmicos de enfermagem, tais achados vão ao encontro de toda a trajetória histórica da profissão de ser majoritariamente feminina^(10)^. Mas vale ressaltar que, mesmo sendo uma profissão majoritariamente feminina, consideram que nos últimos anos há uma tendência para o aumento da participação masculina na categoria^(10)^.

As pessoas utilizam o mundo virtual como um instrumento para conversar, se relacionar e interagir, seja com indivíduos que já conhecem ou com aqueles que passam a conhecer pelas redes sociais, mas não possuem convívio presencial. As oportunidades oferecidas pelo mundo digital são impressionantes, porém é importante não absolutizá-las teoricamente ou praticamente, e, para isso, é preciso de pensamento e tempo, e pensar leva tempo^(11)^.

O ciberespaço permite uma interação social, o compartilhamento de valores, saberes, objetivos, oportunidades, conectando as pessoas, proporcionando novas amizades e permitindo uma rápida mobilização entre os indivíduos em qualquer lugar do mundo. Atualmente, o uso desse meio pelos profissionais de saúde propicia um novo contexto ético na formação e na prática assistencial, pois a maioria das pessoas possui câmera fotográfica, que pode facilmente registrar imagens e vídeos de situações vivenciadas por pacientes, colegas de trabalho ou na prática acadêmica dentro das instituições de saúde^(12)^.

O uso do ciberespaço está aumentando gradativamente entre estudantes e profissionais de enfermagem, porque é um espaço de interação social, onde informações da área da saúde podem ser obtidas e compartilhadas, pois facilitam a comunicação e permitem a troca de opiniões e experiências de casa ou do trabalho. Além disso, facilitam a atualização, por ser uma ferramenta fundamental para os periódicos biomédicos divulgarem seus artigos científicos para pesquisadores e leitores^(13)^.

Os acadêmicos de enfermagem, que são jovens na sua maioria, utilizam o mundo virtual como uma ferramenta para produzir e postar suas ideias, pensamentos e promover muitas maneiras de interação e socialização^(14)^. Dentro dessa utilização, podem estar sendo exibidas fotografias de pacientes durante uma atividade acadêmica nos serviços de saúde, como foi identificado em pesquisa realizada com estudantes universitários da saúde de Manaus, sem solicitar permissão para isso^(12)^. E em pesquisa realizada no México, profissionais de enfermagem não estão respeitando a confidencialidade dos pacientes ao publicar imagens e informações^(13)^.

Considerando tais informações, é preciso lançar mão da ética do cuidado, que são aspectos da ética e da moral aplicados na saúde, como a empatia, a autonomia (do profissional e do paciente), o compromisso com os direitos e deveres humanos, a cidadania e o multiculturalismo^(15)^.

As relações estabelecidas no mundo virtual atravessam as fronteiras das nações e, mesmo assim, os direitos e deveres nesse ambiente não podem ser isolados dos direitos e deveres do mundo físico^(16)^. As regras de boa convivência e segurança que lançamos mão na vida real também precisam ser utilizadas no mundo virtual, ou seja, é preciso bom senso e pensar varias vezes antes de compartilhar qualquer informação no mundo virtual.

Assim, os profissionais de enfermagem precisam conservar e definir uma atitude ética ao publicar ou compartilhar informações relacionadas à saúde dos pacientes^(13)^. Logo, a ética mostra-se um desdobramento das interações sociais das pessoais mais próximas, das quais se estabelecem laços afetivos, e o comportamento moral teria como alicerce a reciprocidade presente, principalmente, nas relações de cuidado^(17)^.

A privacidade no ciberespaço não tem sido mais uma prioridade entre as pessoas, pois os profissionais de saúde, incluindo os estudantes, mesmo com autorização, podem expor desnecessariamente informações e imagens dos pacientes^(12)^. Consequentemente, não consideram os critérios estabelecidos pelo Código de Ética da Enfermagem e resolução do COFEN.

Além disso, o mundo virtual não é fechado e nem privativo, e uma foto pode ganhar o mundo em segundos ao ser visualizada por milhões de pessoas, pois não se está protegido atrás de uma tela de computador. Logo, é possível considerar e imaginar um futuro em que a diferença entre o real e o digital, como uma possível perspectiva de vida, seja percebida como confusa^(11)^. Desse modo, os dados divulgados no mundo virtual envolvem um risco, porque qualquer um pode baixar ou compartilhar as informações e, consequentemente, permanecer *online* por tempo indeterminado; assim, as informações que são compartilhadas ou publicadas podem causar um problema ético ou legal^(18)^.

O acadêmico de enfermagem, durante a sua formação educacional, adquire uma identidade profissional que carrega valores e comportamentos e que garante a confiança dos pacientes; logo, é preciso ter cautela com as informações que são publicadas no mundo virtual para não criar uma imagem de trabalho negativa^(18)^.Tais valores e comportamentos podem girar em torno do cuidado recebido, principalmente o da infância, envolvendo sentimentos e afetos que podem servir de base para o que se interpreta como “certo” ou “errado” nas relações humanas, o que pode ser considerado com “importa-se com” ^(17)^.

É preciso não só pensar no local de trabalho, mas também nas relações interpessoais entre os colegas, pois uma foto, um vídeo ou um comentário compartilhado no mundo virtual pode ocasionar um conflito entre eles e dificultar a convivência cotidiana e, com isso, afetar o andamento da assistência e até mesmo ocasionar a demissão dos mesmos. Por isso, os estudantes precisam tomar consciência desde a graduação da importância em respeitar as regras de boa convivência também no ciberespaço, para que não haja desavenças entre os colegas de trabalha e isso afete o cuidado ofertado.

Quando o compartilhamento de ideias, valores e pensamentos no mundo virtual abre espaço para discursos de ódios e intolerância, tendo como justificativa a liberdade de expressão e o livre arbítrio, não pode ser considerado argumento. Tais ofensas definem o *bullying*, que já afetou 27% de crianças e jovens entre 9 e 17 anos no país nos últimos 12 meses^(14)^.Conteúdos ofensivos, com o único intuito de atingir grupos específicos, como minorias sociais, são sempre direcionados à inferiorização da pessoa, raça, gênero, etnia, nacionalidade, religião, orientação sexual, ou outro aspecto, no mundo virtual, caracterizando o *cyberbullying* ^(18)^.

O *cyberbullying* está relacionado ao uso das tecnologias de comunicação e informação para ameaçar, humilhar ou praticar qualquer ação com má intenção a alguém, podendo afetar a convivência, o bem estar mental das vitimas e os seus direitos^(14)^.Geralmente, seu objetivo é afetar o psicológico e o emocional do individuo, mas pode chegar à violência física em si.

Por mais que a pessoa esclareça o seu comportamento e o motivo que o levou a divulgar esse discurso de ódio e intolerância, é algo difícil de ser esquecido. Ainda mais com a velocidade com que se propagam informações no mundo virtual, o assunto poderá ser sempre lembrado e incentivar novas reações.

Assim, é preciso possuir uma boa gestão do que se compartilha ou posta no ciberespaço, para que se evitem constrangimentos ou até mesmo que o comportamento afete diretamente a sua profissão e/ou seu ambiente de trabalho e/ou o cargo que se exerce no momento e/ou a sua vida pessoal. É preciso verificar a veracidade das informações compartilhadas, principalmente aquelas que envolvem assuntos de saúde, pois foram identificados perfis que publicam informações falsas ou que vêm de fontes não confiáveis, uma situação que pode causar riscos à saúde dos seguidores dessas páginas^(13)^.

Dessa forma, é necessário formar profissionais de enfermagem que possuam um compromisso ao divulgar informações digitais, que envolve algumas considerações regulatórias, legais e morais contra a desonestidade acadêmica, como omissão de citações, invenção de conteúdos, autoplágio, duplicação de informações, cópias sem autorização de código-fonte e falsa autoria. Tais conteúdos devem ser enfatizadas como eixo fundamental para a profissão^(16,18)^.

Nesse sentido, as pessoas e a sociedade em geral precisam ser protegidas legalmente, mas não de forma excessiva e paternalista, nem sujeitas a controle total sem qualquer tipo de acordo legal sobre as necessidades e limites de tais medidas^(19)^. As pessoas podem fazer uso da obrigação moral, que envolve o reconhecimento de outros indivíduos como semelhantes. No que diz respeito à necessidade de dar e receber cuidado, aplicam-se de maneira indireta os reais sentimentos pelo outro^(17)^.

Desse modo, o guia pode levar os acadêmicos de enfermagem a melhor entender esses exemplos, ainda na graduação, para evitar que tais ações sejam replicadas quando eles estiverem no mercado de trabalho (e também nas suas vidas pessoais). Portanto, o profissional de enfermagem deve ser dotado de sólida experiência ética e tecnológica, que deve começar na fase universitária, com o objetivo de respeitar os princípios éticos e, assim, permitir melhorara capacidade de prevenir e resolver conflitos éticos na execução de sua função^(18)^.

Nesse sentido, essa prática profissional e acadêmica pode ser guiada pela ética do cuidado, que é possível ser representada pela síntese dos valores morais aplicados à prática do cuidar, abrangendo as virtudes morais, a prudência, a justiça, o respeito à autonomia, a liberdade e a democracia^(15)^.

### Limitações do estudo

O estudo tem como limitação as entrevistas terem sido realizadas somente em uma universidade e com apenas a turma do primeiro período, o que limita uma análise da realidade brasileira. A inclusão de outras universidades poderá ampliar o escopo de análise do comportamento dos estudantes de enfermagem no ciberespaço.

### Contribuições para a áreas da enfermagem, saúde ou políticas públicas

O presente estudo leva os estudantes a terem cuidado com aquilo que é postado e compartilhado no ciberespaço, pois as informações podem ser inverdades, sem nenhum controle e sem conseguir identificar a fonte original. Desse modo, precisam parar para refletir e analisar com calma e dentro dos preceitos éticos o que estão lendo, postando e compartilhando nas suas redes virtuais.

## CONSIDERAÇÕES FINAIS

Os acadêmicos de enfermagem utilizam o ciberespaço para facilitar a comunicação, interagir, estudar, obter informações, guardar fotos e arquivos, mas também retratam que ações ruins podem ser identificadas, como a *fake news* e o *cyberbullying*.

Nesse sentido, faz-se importante incluir e/ou reforçar no currículo do curso de enfermagem como melhor usar o ciberespaço, destacando os princípios éticos e legais, bem como o impacto sobre sua vida profissional e pessoal. Assim, o guia de orientação poderá ser uma importante ferramenta para nortear essas ações junto às instituições de ensino e de saúde.
